# Future of Dentistry

**Published:** 1862-11

**Authors:** J. Taft


### THE FUTURE OF DENTISTRY.

BY J. TAFT.

PERHAPS no one would venture to attempt to predict what shall be the future attainments of the science and practice of the dental profession. It is not easy to decide in what particulars the greatest and most rapid progress will be made. There are so many things leading men astray from that which should be the paramount object of life's action, that it is difficult to arrive at a just conclusion in regard to what attainments will be made, without taking into consideration these diverting circumstances.

We do not propose to write a chapter of prophesies, but simply to make a few statements in regard to what the future of our profession should be, and what it will be, under proper guidance. And first, it will not be, as has been declared by some, whatever is demanded by those requiring the aid of the dentist, at least until they have a just appreciation of the teeth, and a proper knowledge of what is necessary for their preservation. This knowledge must radiate from the dental profession.

Neither will it be that which would be suggested by the great proportion of the profession in its present status, but it will be an emanation from the profession, after having been subjected to a more thorough cultivation. As in general medicine it will not be the prominent idea, to repair lost organs by artificial substitutes, nor to patch and repair parts that have become marred, injured or partially destroyed, by disease. The prominent idea should and will be, to order such an arrangement of things as will ward off disease, and prevent the attack of the enemy. This every one will admit would be far better than to suffer the gates to be forced, and the walls battered down, and then attempt to build up the walls with inferior and incompatible materials, while the enemy too often remains within.

We will have a profession whose training, study and investigation will not be confined to the oral cavity and immediate appendages, but must embrace the entire human system, at least so far as the teeth may be at all influenced by the condition of its parts. More attention must be given to the structure and development of the teeth, and to the conditions and circumstances that modify these; this field, alas! is permitted, for the most part, to remain a barren, uncultivated waste, yet it is a soil the cultivation of which would result in the grandest productions.

By enlightened and judicious management during the time of development of the teeth, they might be rapidly changed for the better, so that in every generation a decided improvement would be apparent. In order, however, to accomplish much in this direction, the dentist must understand well the whole subject; he must then have the coöperation and obedience of well informed and confiding patrons.

The attention of the profession in the future must be directed more than hitherto to the causes of dental caries, and to their methods of operation, and to the most efficient means of arresting their progress. The truth in regard to the great majority of operators is, that in regard to the natural teeth, they simply endeavor to repair the breaches already made, without caring to inquire whether the enemy is not still within the citadel, or under its walls, continuing to batter away upon them. Of course, under such circumstances, the breaches he may repair are likely to be broken again, and other points attacked and broken down; the true method would be to find out the hiding place of the enemy; find out his character and strength, and then drive him out, or break his power.

Few seem to care what did the mischief, so they may make a temporary repair, and secure a fee. This in too many cases is the ruling power; can we operate and get the fee—there the matter ends, so far as the welfare of the patient is concerned; this is a circumscribed effort to do good, it is acting from the lowest motive.

He who performs the most perfect operations—for instance in filling teeth—in the manipulative point of view, and disregards conditions, will find ere long that his best efforts have failed, under the influence of the same agents that began the destructive work. And certainly he who performs poor operations, and goes no farther, can hope for very little indeed.

Now, in respect to all this, in the future a change will come; dentists will be more thoroughly educated; their education embrace a far wider range; they will take a far deeper hold upon all they attempt, and this must be instilled into the minds of the people, at least so far as it is necessary to effect a concurrence of action.

Dentists will learn to act from higher motives than the dollar; they will find there is a higher reward for the highest effort to ward off disease and protect from its ravages.

Perhaps teeth will be filled for a great while to come, but under a proper order of things, it should become less and less year by year, till by and by, there would only occasionally be such a thing as a filled tooth, and such things as sets of artificial teeth be but seldom, if at all found. Were there a proper appreciation of the teeth, they would be as scrupulously cared for as the eyes or the ears, for the welfare of the whole economy depends far more upon the teeth than upon these. The remark is often made, upon losing one or more teeth, I have more teeth left. Every body will say that none but a fool would make such a remark about an eye or an ear. Such things show a want of proper appreciation of the teeth; this, however, will grow up in that good time coming.

